# Serovar‐Specific Antimicrobial Resistance and Virulence Profiles of *Salmonella enterica* From Poultry in Bangladesh

**DOI:** 10.1002/mbo3.70091

**Published:** 2025-10-22

**Authors:** Najmun Nahar Popy, Mohammad Ferdousur Rahman Khan, Md. Saiful Islam, Limon Biswas, Layla Yasmin, Mahbubul Pratik Siddique, Marzia Rahman, Md. Bahanur Rahman

**Affiliations:** ^1^ Department of Microbiology and Hygiene Bangladesh Agricultural University Mymensingh Bangladesh; ^2^ Department of Animal Science University of California – Davis Davis USA

**Keywords:** Bangladesh, multidrug resistance, nontyphoidal *Salmonella*, *Salmonella* serovars, virulence determinants

## Abstract

*Salmonella enterica* is a major foodborne pathogen that poses significant risks to public health and poultry production. This study aimed to investigate the distribution, serovar types, virulence genes, and antimicrobial resistance (AMR) profiles of *S. enterica* isolated from chickens in Bangladesh. A total of 250 samples from broiler (*n* = 100), layer (*n* = 100), and Sonali (*n* = 50) chickens were collected across four districts in Bangladesh and analyzed using polymerase chain reaction (PCR) and MALDI‐TOF for identification and serotyping. The presence of virulence and AMR genes was assessed via PCR, while phenotypic resistance was determined using the disk diffusion method. In PCR, *S. enterica* was detected in 14.4% (36/250) of the samples, with significantly higher prevalence in layers compared with broilers and Sonali. Among the isolates, 61.1% (22/36) were identified as serovar Typhimurium, 27.8% (10/36) as Enteritidis, and 11.1% (4/36) remained untyped. All isolates harbored the *invA*, *stn*, *sivH*, and *lpfA* virulence genes, while *hilA* and *spvC* were detected in 97.2% and 63.9% of the isolates, respectively. High phenotypic resistance was observed to sulfamethoxazole (94.4%), sulfonamide (91.7%), nitrofurantoin (55.5%), and ampicillin (52.8%), with 86.1% of isolates classified as multidrug‐resistant. Genotypically, resistance genes *aadA2* and *SUL1* were most common (94.4%), followed by *bla*_CMY‐9_ (75%) and *bla*_SHV_ (52.8%), with variations across serovars and poultry types. These findings highlight the high burden of antimicrobial‐resistant *S. enterica* serovars in Bangladeshi poultry and underscore the need for continuous surveillance and prudent antimicrobial use.

## Graphical abstract

This study provides an updated assessment of *Salmonella enterica* in poultry farms across Bangladesh, revealing a 14.4% prevalence with diverse serovars, virulence determinants, and antimicrobial resistance patterns. The findings highlight farm‐level poultry as a reservoir of resistant and potentially pathogenic *Salmonella*, underscoring the risks to both animal and public health.

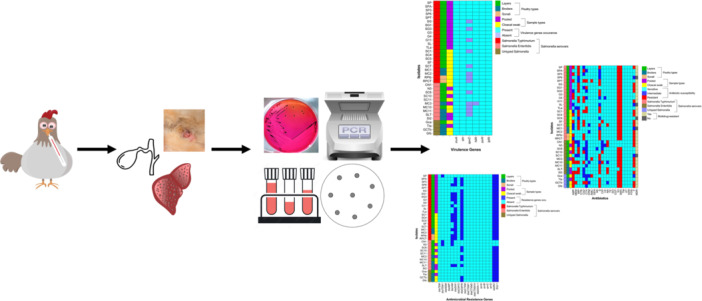

## 1 Introduction

Nontyphoidal *Salmonella* (NTS) is a major global public health concern and ranks among the leading causes of foodborne illnesses worldwide (Malkiely et al. 2025; Intuy et al. 2024). The majority of NTS outbreaks are associated with the consumption of contaminated animal‐derived food products, particularly poultry and poultry products, which represent one of the most widely consumed and economically accessible protein sources globally (X. Jiang et al. 2025; Polat et al. 2024). A recent report from the United States Department of Agriculture (USDA 2023) attributed 23% of NTS infections in the United States to poultry consumption. Globally, NTS is responsible for an estimated 59,000 deaths and nearly 4 million disability‐adjusted life years lost annually (Sun et al. 2024).

There are over 2600 known *Salmonella* serovars, with *Salmonella enterica* serovars Typhimurium and Enteritidis being the most frequently associated with human infections (Y. Hu et al. 2022; Hong et al. 2023). NTS infection poses a greater threat to children and immunocompromised individuals, often leading to bacteremia and severe outcomes, particularly in patients with underlying conditions (da Silva et al. 2024). In Bangladesh, patients with NTS‐related bacteremia and diarrhea commonly exhibit comorbidities and have a higher case fatality rate than those with typhoidal infections (Shahunja et al. 2015).

In poultry, *Salmonella* infection disrupts intestinal microbiota, damages the mucosal barrier, and leads to inflammation, diarrhea, and growth retardation (Z. Hu et al. 2023). The pathogen invades through the compromised intestinal barrier and disseminates to internal organs such as the liver and gallbladder via the reticuloendothelial system (Rana et al. 2021). *Salmonella* Enteritidis is known to cause systemic disease and high mortality in young birds, while adult birds often act as asymptomatic carriers (Shaji et al. 2023). Similarly, neonatal chicks are highly susceptible to *Salmonella* Typhimurium, which can lead to systemic infection; however, adult birds may harbor the bacteria without exhibiting clinical signs (Sutton et al. 2023).

The pathogenicity of NTS is largely attributed to the presence of virulence‐associated genes that facilitate host invasion, toxin production, adhesion, biofilm formation, and immune evasion (Lu et al. 2022; Rezaei et al. 2022). Central to *Salmonella* virulence are type III secretion systems encoded by *Salmonella* pathogenicity islands (SPIs). SPI‐1 facilitates intestinal invasion and enteritis by delivering effector proteins, whereas SPI‐2 enables intracellular survival and systemic spread by supporting replication within macrophages (Tang et al. 2023; Billah and Rahman 2024; Chu et al. 2024).

Antimicrobial resistance (AMR) has been declared one of the top 10 global public health threats by the World Health Organization (Harun et al. 2024). Due to its widespread presence in food animals and the environment, NTS is a significant contributor to the emergence and spread of AMR (Zavari et al. 2025). The inappropriate use of antimicrobials in agriculture, especially for growth promotion and prophylaxis, has accelerated the emergence of resistant NTS strains, which can be transmitted to humans through the food chain (Hasegawa et al. 2024; Zhao et al. 2023). As a result, AMR *Salmonella* has been listed among the WHO's global priority pathogens (Z. Jiang et al. 2021).

Multidrug‐resistant (MDR) NTS has emerged as a critical public health concern, with prevalence varying globally but showing increasing trends, particularly in South and Southeast Asia. Reported MDR rates include Thailand (69%), Taiwan (53.8%), India (68%), and Bangladesh (94%), as well as sub‐Saharan Africa (77.2%–87.4%) (Starkova et al. 2025). MDR NTS infections are associated with higher mortality, prolonged hospital stays, increased bloodstream infections, and greater healthcare costs (Yin et al. 2022). Resistance to critically important antimicrobials, such as third‐generation cephalosporins and fluoroquinolones, first‐line treatments for severe NTS infections, has further intensified the clinical burden (Piña‐Iturbe et al. 2024).

In Bangladesh, commercial poultry production, including broiler, layer, and Sonali operations, is expanding rapidly in response to growing demand for meat and eggs (Chowdhury et al. 2022). Therefore, monitoring the distribution of *Salmonella* serovars and their AMR patterns in food‐producing animals is essential due to global trade in animal products (Yu et al. 2021). In Bangladesh, NTS remains a major concern in the poultry sector, contributing to substantial economic losses (Hossain et al. 2021). Wet markets serve as reservoirs for virulent NTS strains, facilitating transmission to humans (Siddiky et al. 2022). Serotyping plays a crucial role in tracking epidemiological trends, identifying contamination sources, and understanding virulence and AMR profiles (Bescucci et al. 2022). Addressing the AMR threat requires coordinated surveillance, appropriate antimicrobial use, and effective therapeutic interventions (Ripon et al. 2023).

While several studies in Bangladesh have examined AMR profiles in NTS (Alam et al. 2020; Zamil et al. 2021; Sarker et al. 2021; Uddin et al. 2021; Sultana et al. 2021; Das et al. 2022), only a few have investigated virulence gene distribution (Siddiky et al. 2021, 2022). Apart from a single study focused on diarrheic chickens (Sultana et al. 2021), limited data exist on the zoonotic potential of *Salmonella* in poultry. This study aims to fill that gap by characterizing zoonotic *Salmonella* in clinically suspected chickens in Bangladesh, with a focus on serovar distribution, virulence attributes, and both phenotypic and genotypic AMR profiles.

## 2 Materials and Methods

### 2.1 Ethical Consideration

The methodologies and related protocols used in this study were approved by the Animal Welfare and Experimentation Ethics Committee (AWEEC) of Bangladesh Agricultural University, under approval number AWEEC/BAU/2022 (21).

### 2.2 Study Area Selection

**Figure 1:**
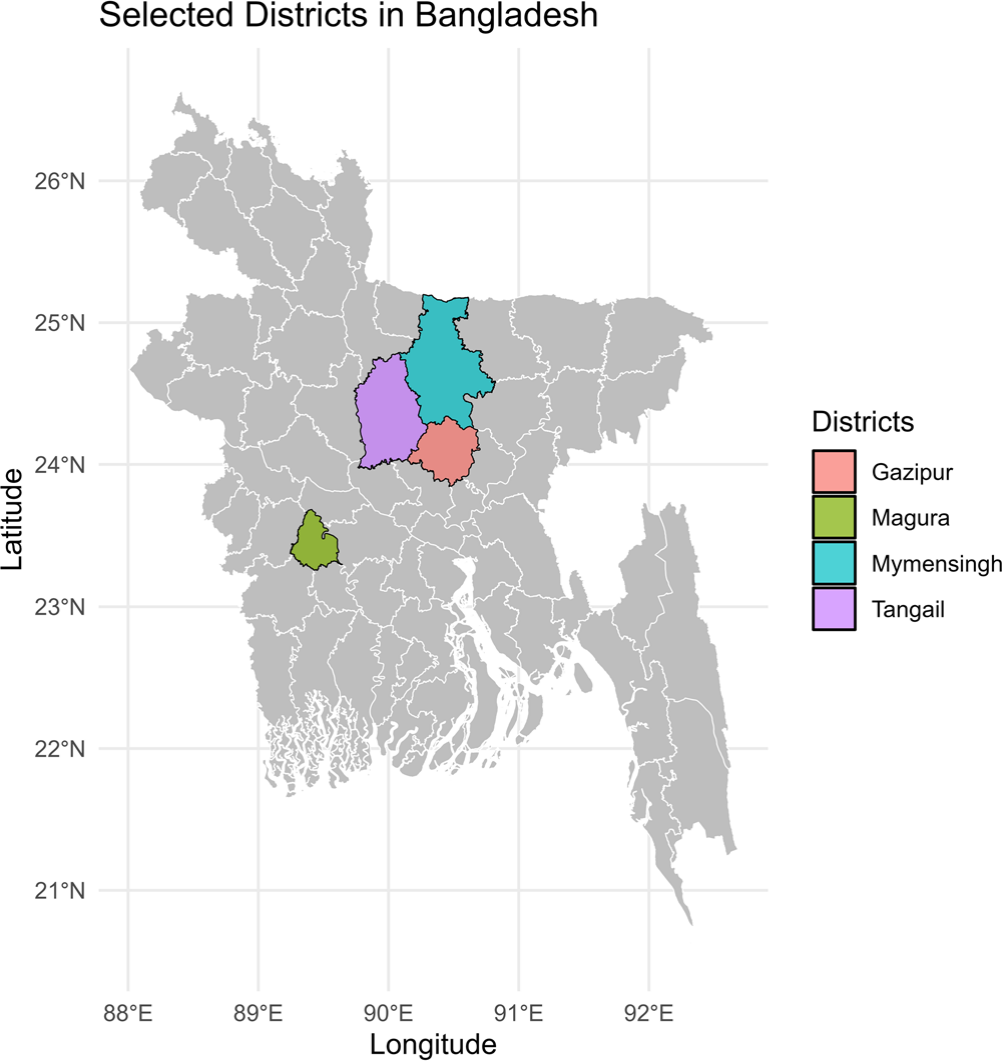
Study districts in Bangladesh, Gazipur, Magura, Mymensingh, and Tangail were selected for poultry sampling in this study.

This cross‐sectional study was conducted from March 2021 to June 2023 in four different districts in Bangladesh, including Mymensingh (24.7539° N, 90.4073° E), Gazipur (24.0958° N, 90.4125° E), Tangail (24.3917° N, 89.9948° E), and Magura (23.4290° N, 89.4364° E) (Figure 1). These districts were purposefully selected due to their intensive poultry farming practices involving broiler, layer, and Sonali chickens, their central role in the country's poultry production network, and the availability of veterinary and laboratory infrastructure necessary for sample collection and analysis. Moreover, Sonali chickens, a popular dual‐purpose crossbreed in Bangladesh (a cross between Fayoumi cocks and Rhode Island Red hens), are widely reared for both meat and egg production. They were analyzed separately in this study due to their distinct management practices, longer rearing period compared with commercial broilers, and their unique role in the country's poultry production system.

### 2.3 Sample Size Calculation

The sample size is calculated based on the prevalence of *Salmonella* spp. found in previous studies in Bangladesh. The prevalence of *Salmonella* in cloacal swabs of diseased layer chickens was 37.03% (Sultana et al. 2021). The sample size for this study is calculated based on an assumed prevalence of 37%, a 95% confidence interval (CI_95_), and an accepted error precision of 6% (Daniel 1999). The sample size is calculated by using “sample size calculator version 1.0.01 and formula for prevalence studies” (Daniel 1999; Naing et al. 2006). The formula of sample size calculation, *n* = *Z* ^2^ *pq*/*d* ^2^ was used, where *n* is the desired sample size, *Z* the normal standard deviation (1.96 at 95% CI), *p* the prevalence (37% or 0.37), *q* = (1 − *p*) = (1–0.37) = 0.63, *d* = Precision (6% or 0.06). So, *n* = (1.96)^2^ × 0.37 × 0.63/(0.06)^2^ = 249. Therefore, we decided to collect 250 samples aseptically in this study.

### 2.4 Sample Collection and Processing

A total of 250 chickens (170 live and 80 dead) were selected from different farms (*n* = 15). The distribution of samples was as follows: 115 from Mymensingh, 81 from Gazipur, 24 from Tangail, and 30 from Magura. The number of birds per farm depended on flock size and availability of clinically affected or freshly dead birds at the time of sampling. Live birds were chosen based on observable clinical signs suggestive of *Salmonella* infection, including depression, diarrhea, ruffled feathers, stunted growth, and decreased egg production. Dead birds were included if they showed gross postmortem lesions during necropsy, such as hepatomegaly, hepatic congestion, friability of the liver, and enlarged gallbladders. From each live bird, a cloacal swab was collected using a sterile cotton swab, while pooled gallbladder and liver tissues (PGLT) were obtained from each dead bird during necropsy. Two experienced veterinarians performed all sample collections.

Immediately after collection, samples were transferred to the laboratory following a cool chain. Cloacal swabs and PGLT samples were each placed into Whirl‐Pak bags containing 20 mL of buffered peptone water (HiMedia, India). After mixing the samples with a homogenizer (Infitek, Shandong, China), they were incubated at 37°C for 18 h. Cultured samples (1 mL) were added to 9 mL of Rappaport‐Vassiliadis (RV) medium (HiMedia, India) to achieve a 1:10 dilution, and incubated at 42°C for 24 h.

### 2.5 Detection of *Salmonella* Isolates and *S. enterica* Serovars

Ten µL of RV broth cultures were streaked on Xylose Lysine Deoxycholate agar (XLD, Himedia, India) and incubating them at 37°C for 24 h. Presumptive *Salmonella* isolates were determined by their colony characteristics (black centered colonies on XLD agar plates), Gram staining, and biochemical tests (Crump et al. 2015). Finally, presumptive *Salmonella* isolates were confirmed by the matrix‐assisted laser desorption/ionization time‐of‐flight mass spectrometry (MALDI‐TOF‐MS) (Al‐Hindi et al. 2023).

*S. enterica* isolates and their serovars were evaluated by polymerase chain reaction (PCR) assays. To do that, genomic DNA was extracted from *Salmonella*‐positive isolates using the boiling technique (Ievy et al. 2022). *S. enterica* was identified using a species‐specific PCR assay that targeted the *bcfC* gene (Table S1). Moreover, PCR‐specific *flic* and *sdf* genes were used to identify *S. enterica* serovar Typhimurium and *S. enterica* serovar Enteritidis, respectively (Table S1).

All PCR amplification was carried out in a final reaction volume of 20 µL, consisting of 2 µL of DNA template, 12 µL of 2X GoTaq Green Master Mix (Promega, Madison, WI, USA), 1 µL each of forward and reverse primers, and 4 µL of nuclease‐free water. PCR products were analyzed by electrophoresis on a 1.5% agarose gel prepared in 1× tris‐borate‐ethylenediamine tetra‐acetic acid buffer, stained with ethidium bromide (0.5 µg/mL), and visualized under ultraviolet illumination. DNA bands were visualized using the ChemiDoc Imaging System (Bio‐Rad, Hercules, CA, USA). *S. enterica* Typhimurium ATCC 14028 and *S. enterica* Enteritidis ATCC 13076 were used as positive controls, while phosphate‐buffered saline was used as a PCR‐negative control instead of genomic DNA.

### 2.6 Molecular Detection of Virulence Genes in *Salmonella* Species

Six virulence‐associated genes were screened in *Salmonella* isolates to evaluate their pathogenic potential. These included *invA* (invasion gene), *spvC* (plasmid‐encoded gene), *stn* (enterotoxin gene), *lpfA* (fimbrial gene), *hilA* (regulatory protein gene), and *sivH* (effector protein gene). Each gene corresponds to a distinct virulence mechanism, such as host cell invasion (*invA*), systemic infection (*spvC*), toxin production (*stn*), adhesion via fimbriae (*lpfA*), transcriptional regulation of invasion genes (*hilA*), and secretion of effector proteins (*sivH*) (Table S2) (Arkali and Çetinkaya 2020; Farhat et al. 2023; Lozano‐Villegas et al. 2023; Ndlovu et al. 2023; Siddiky et al. 2022; Yulian et al. 2020). The detection of these genes was performed using gene‐specific primers via conventional PCR.

### 2.7 Antimicrobial Susceptibility Testing of the *Salmonella* Isolates

The antimicrobial susceptibility of *Salmonella* isolates was phenotypically assessed using the Kirby–Bauer disc diffusion method on Mueller–Hinton agar (HiMedia, India), in accordance with the guidelines of the Clinical and Laboratory Standards Institute (Clinical and Laboratory Standards Institute 2024). Zone diameters were interpreted as susceptible (S), intermediate (I), or resistant (R) in accordance with CLSI breakpoints (Clinical and Laboratory Standards Institute 2024). A total of 24 antimicrobial agents (HiMedia, India), representing 12 distinct antimicrobial classes, were evaluated:

- *Aminoglycosides:* gentamicin (10 µg), amikacin (30 µg), and streptomycin (10 µg).
- *Penicillins:* ampicillin (25 µg).
- *Cephalosporins:* ceftriaxone (30 µg), cefotaxime (30 µg), ceftazidime (30 µg), and cefepime (30 µg).
- *Carbapenems:* imipenem (10 µg) and meropenem (10 µg).
- *β‐Lactamase Inhibitor Combinations:* amoxicillin‐clavulanate (30 µg).
- *Quinolones:* ciprofloxacin (5 µg), levofloxacin (5 µg), and nalidixic acid (30 µg).
- *Tetracyclines:* tetracycline (30 µg) and doxycycline (30 µg).
- *Folate Pathway Inhibitors:* sulfonamides (300 µg), sulfamethoxazole (100 µg), trimethoprim (2.5 µg), and trimethoprim‐sulfamethoxazole (25 µg).
- *Phosphonic Acids:* fosfomycin (50 µg).
- *Macrolides:* azithromycin (30 µg).
- *Monobactams:* aztreonam (30 µg).
- *Nitrofurans:* nitrofurantoin (300 µg).

*Escherichia coli* ATCC 25922 was used as the quality control strain. Isolates exhibiting resistance to at least one agent in three or more antimicrobial classes were classified as MDR (Magiorakos et al. 2012). The Multiple Antibiotic Resistance (MAR) index, a reliable tool for assessing exposure to high‐risk antibiotic contamination sources, was calculated for each isolate by dividing the number of antibiotics to which the isolate was resistant by the total number of antibiotics tested (Ayandele et al. 2020). An MAR index value greater than 0.2 indicates contamination originating from environments where antibiotics are frequently used (Titilawo et al. 2015).

### 2.8 Detection of Antibiotic Resistance Genes by Molecular Approach

A broad panel of AMR genes was screened in the isolates using conventional PCR (Table S3). These included β‐lactamase genes (*bla*_TEM_, *bla*_TEM‐1_, *bla*_SHV_, *bla*_CMY‐2_, *bla*_CMY‐9_, *bla*_CTX‐M_, *bla*_CTX‐M‐1_, *bla*_CTX‐M‐2_, *bla*_CTX‐M‐14_, and *bla*_OXA_), plasmid‐mediated carbapenem resistance genes (*bla*_NDM_, *bla*_IMP_, and *bla*_VIM_), quinolone resistance determinants (*qnrA*, *qnrB*, *qnrC*, and *qnrS*), a sulfonamide resistance gene (*SUL1*), and an aminoglycoside resistance gene (*aadA2*). The PCR reaction mixture composition and product visualization methods were consistent with those used for detecting *Salmonella* isolates.

### 2.9 Statistical Analysis

All data were compiled and analyzed using Microsoft Excel (Excel 2013, Los Angeles, CA, USA) and R software (version 4.3.2). The prevalence of *S. enterica* isolates and their serovars, virulence genes, and AMR patterns was expressed as percentages with corresponding 95% CIs (CI_95_). Pearson's Chi‐square test or Fisher's exact test, as appropriate, was used to assess significant differences in *S. enterica* prevalence, virulence gene distribution, and resistance profiles across sampling sites, bird types, sample types, and serovar categories. A *p‐*value of < 0.05 was considered statistically significant.

## 3 Results

### 3.1 Distribution of *S. enterica* Isolates and *S. enterica* Serovars

In PCR, *S. enterica* isolates were detected in 14.4% (36/250, CI_95_: 10.6–19.3) of the samples. There was a significant variation in the detection rate of *S. enterica* isolates among different sampling sites, with Gazipur district (28.4%) having the highest prevalence, followed by Magura (16.7%), Mymensingh (7%), and Tangail (0%). Within bird types, the prevalence of *S. enterica* isolates was higher (*p* < 0.05) in layers (25%) than in broilers (9%) and Sonali (4%). On the other hand, *S. enterica* isolates were detected at higher levels (*p* < 0.05) in pooled liver tissue and gallbladder swab samples of dead birds (23.8%) than in cloacal swab (10%) samples of live birds (Table 1).

**Table 1:** Prevalence of *Salmonella enterica* isolates in chickens in Bangladesh.

Variables	Categories	No. of samples	No. of positive isolates (%)	95% CI (%)	*p* value
Locations	Mymensingh	115	8 (7^a^)	3.6–13.1	< 0.001
	Tangail	24	0 (0^a^)	0.0–13.8	
	Gazipur	81	23 (28.4^b^)	19.7–39.0	
	Magura	30	5 (16.7^a,b^)	7.3–33.6	
Bird types	Layers	100	25 (25^a^)	17.6–34.3	< 0.01
	Broilers	100	9 (9^b^)	4.8–16.2	
	Sonali	50	2 (4^b^)	0.7–13.5	
Sample types	Cloacal swabs	170	17 (10^a^)	6.3–15.4	0.006
	Pooled liver tissue and gallbladder swabs	80	19 (23.8^b^)	15.8–34.1	

*Note:* Values with different superscripts (a, b) within the same variable differ significantly (*p* < 0.05). Values sharing the same superscript letter are not significantly different.

Abbreviation: CI = confidence interval.

Among 36 *S. enterica*, 22 (61.1%, CI_95_: 44.9; 75.2) *S. enterica* serovar Typhimurium, 10 (27.8%, CI_95_: 15.9; 43.9) *S. enterica* serovar Enteritidis, and four (11.1%, CI_95_: 4.4; 25.3) untyped serovars were identified. Moreover, 18 (18%, CI_95_: 11.7; 26.7) *S. enterica* serovar Typhimurium were identified in layers (13 in pooled liver tissue and gallbladder swabs and five in cloacal swabs), two (2%, CI_95_: 0.4; 7) from broiler cloacal swabs, and two (4%, CI_95_: 0.7; 13.5) from Sonali cloacal swabs. On the other hand, six (6%, CI_95_: 2.8; 12.5) *S. enterica* serovar Enteritidis were detected in layers (two in pooled liver tissue and gallbladder swabs, and four in cloacal swabs) and four (4%, CI_95_: 1.6; 9.8) in broilers (three in pooled liver tissue and gallbladder swabs, and one in cloacal swabs. In addition, untyped *Salmonella* serovars were detected in 1% (CI_95_: 0.1; 5.4) of layer and 3% (CI_95_: 0.8; 8.5) of broiler samples. No *S. enterica* serovar Enteritidis and untyped serovars were detected in Sonali samples.

### 3.2 Virulence Genes Detection in *S. enterica* Serovars

**Figure 2:**
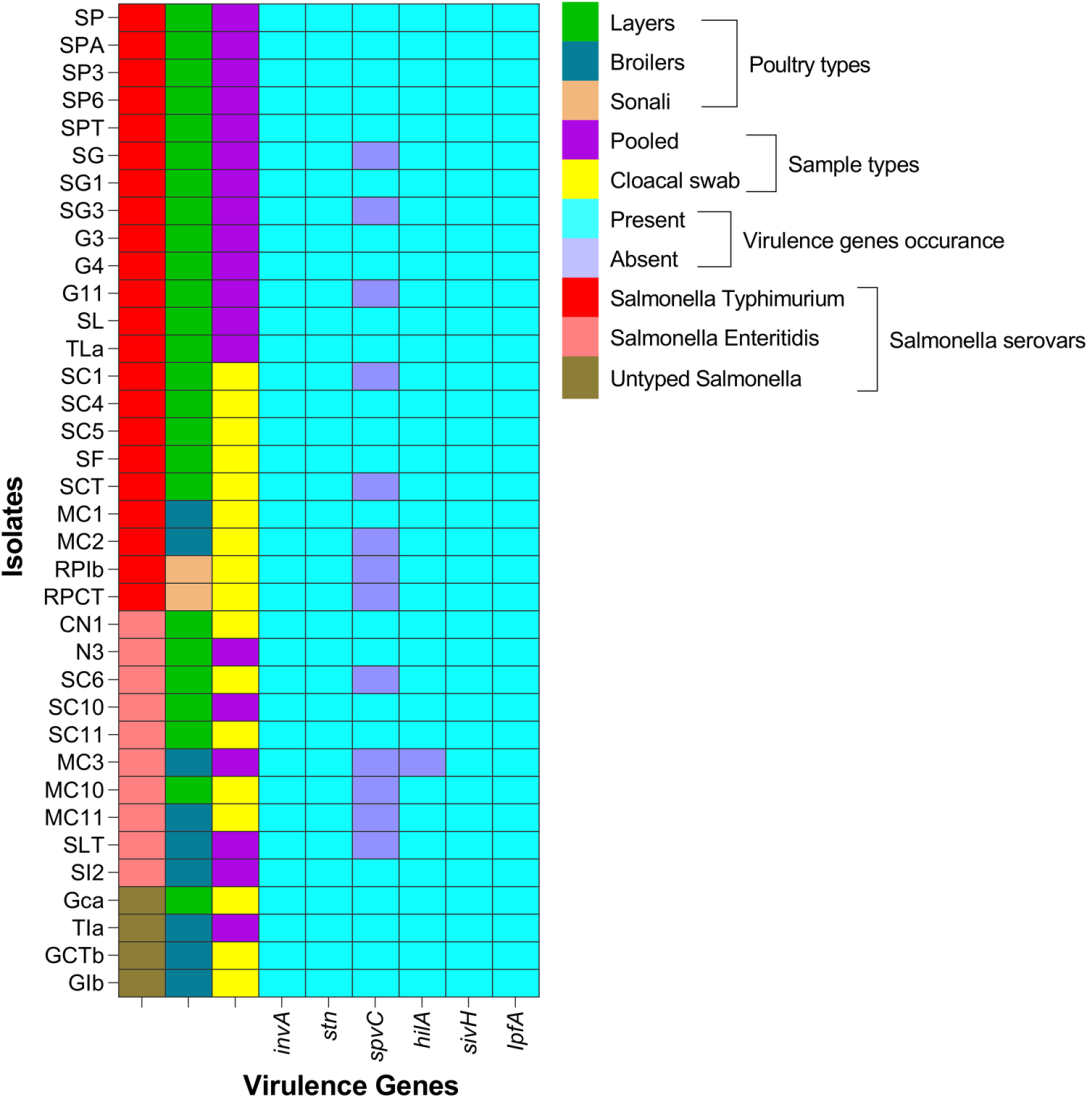
Virulence profiles of *Salmonella enterica* serovars isolated from chickens in Bangladesh.

In PCR, virulence genes *invA*, *stn*, *sivH*, and *lpfA* were found in all *S. enterica* isolates (CI_95_: 90.4; 100), whereas the other two tested virulence genes, *spvC* and *hilA*, were found in 63.9% (CI_95_: 47.6; 77.5) and 97.2% (CI_95_: 85.8; 99.9) isolates, respectively (Figure 2). There was no significant difference in the prevalence of virulence genes detected in poultry types and *S. enterica* serovar types (Table S4).

### 3.3 AMR Profiles of *S. enterica* Isolates

#### 3.3.1 Phenotypic Resistance Profiles

**Figure 3:**
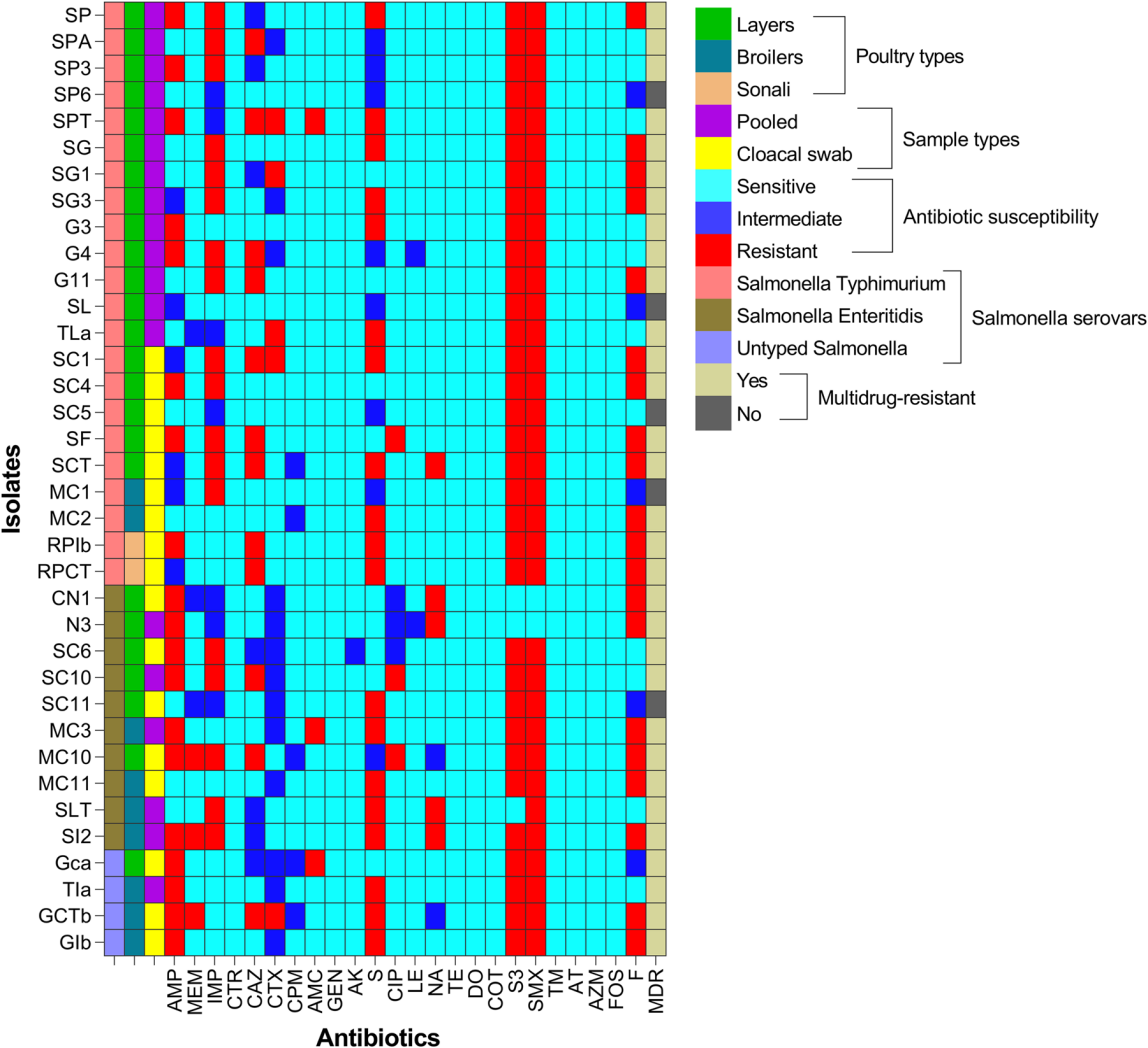
Phenotypic antimicrobial resistance profiles of *Salmonella enterica* serovars isolated from chickens in Bangladesh. AK, amikacin; AMC, amoxicillin‐clavulanate; AMP, ampicillin; AT, aztreonam; AZM, azithromycin; CAZ, ceftazidime; CIP, ciprofloxacin; COT, trimethoprim‐sulfamethoxazole; CPM, cefepime; CTR, ceftriaxone; CTX, cefotaxime; DO, doxycycline; F, nitrofurantoin; FOS, fosfomycin; GEN, gentamicin; IMP, imipenem; LE, levofloxacin; MDR, multidrug resistant; MEM, meropenem; NA, nalidixic acid; S, streptomycin; S3, sulfonamide; SMX, sulfamethoxazole; TE, tetracycline; TM, sulfamethoxazole.

In the disk diffusion test, *S. enterica* isolates were resistance to at least two antimicrobial agents, showing resistance to sulfamethoxazole (94.4%, CI_95_: 81.9; 99.0), followed by sulfonamide (91.7%, CI_95_: 78.2; 97.1), nitrofurantoin (55.5%, CI_95_: 39.6; 70.5), streptomycin and ampicillin (52.8%, CI_95_: 37.0‐68.0), imipenem (50%, CI_95_: 34.5; 65.5), ceftazidime (33.3%, CI_95_: 20.2; 49.7), cefotaxime and nalidixic acid (13.9%, CI_95_: 6.1; 28.7), meropenem and amoxicillin‐clavulanate (8.3%, CI_95_: 2.9; 21.8), and ciprofloxacin (5.6%, CI_95_: 0.9; 18.1). Interestingly, all the *S. enterica* isolates were susceptible or intermediate to other tested antimicrobial agents (Figure 3 and Table S5).

Among poultry sources, Sonali chickens exhibited higher resistance levels, with 100% isolates resistant to ceftazidime, streptomycin, sulfamethoxazole, sulfonamide, and nitrofurantoin. Broiler isolates also showed considerable resistance, particularly to sulfamethoxazole (100%), streptomycin/sulfonamide (88.9%), nitrofurantoin (66.7%), and ampicillin (55.6%); while layer isolates demonstrated lower resistance overall, though resistance to ampicillin (52%) and sulfonamide/sulfamethoxazole (92%) was still prevalent. Resistance patterns also differed by *Salmonella* serovars: Typhimurium isolates showed high resistance to sulfonamide/sulfamethoxazole (100%), imipenem (59.1%), nitrofurantoin (54.5%), and streptomycin (50%). Enteritidis strains were notably resistant to sulfamethoxazole (80%), ampicillin/sulfonamide (70%), and nitrofurantoin (60%), while untyped isolates displayed the highest overall resistance, with 100% resistance to ceftazidime, sulfonamide, and sulfamethoxazole (Table S5).

In total, 86.1% (31/36, CI_95_: 71.3; 93.9) of *S. enterica* isolates were phenotypically classified as MDR, where all the untyped serovars were MDR, followed by Enteritidis (9/10) and Typhimurium (18/22) (Figure 3). Moreover, the MAR indices ranged from 0.08 to 0.33.

#### 3.3.2 Genotypic Resistance Profiles

**Figure 4:**
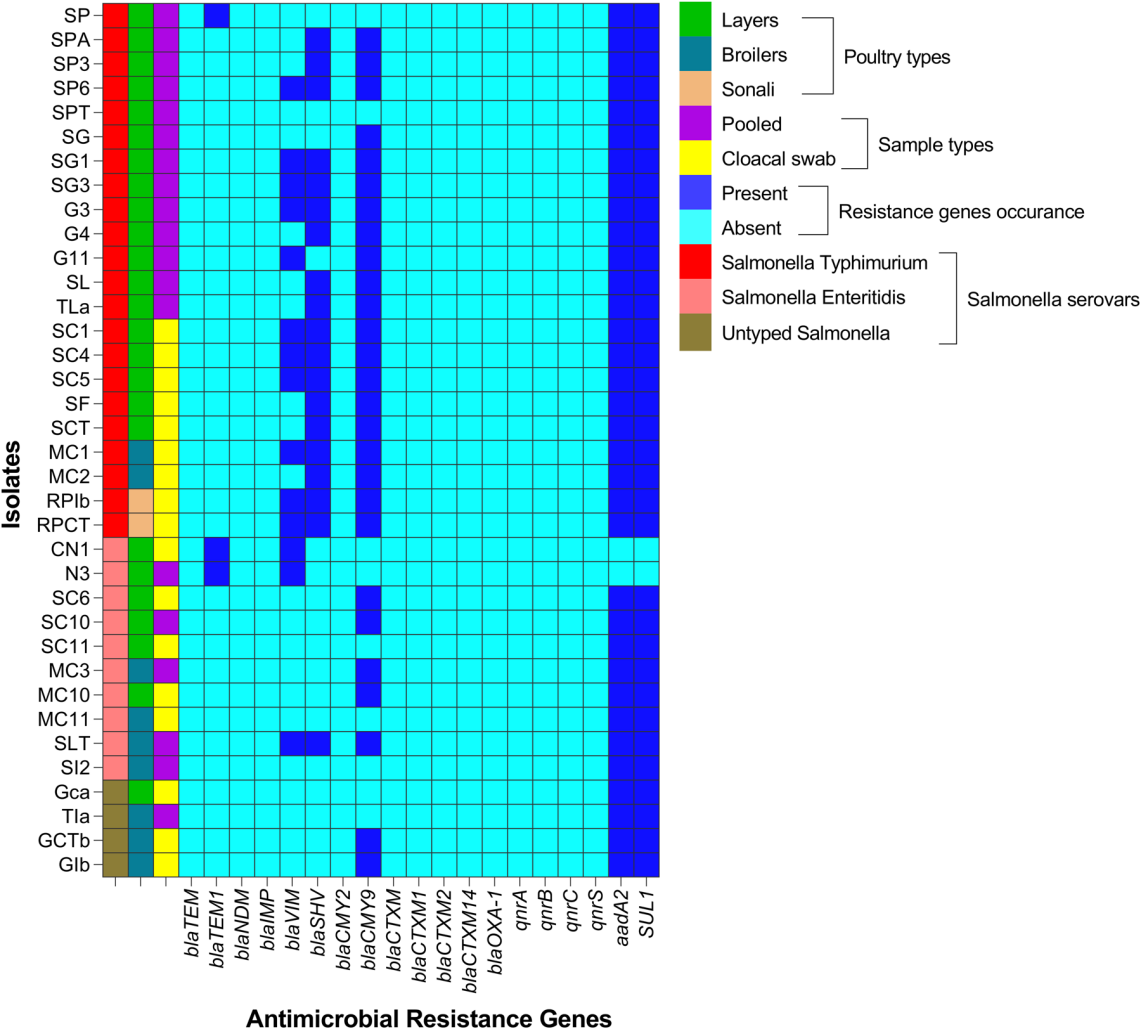
Genotypic antimicrobial resistance profiles of *Salmonella enterica* serovars isolated from chickens in Bangladesh.

In PCR, the most prevalent antibiotic resistance genes among *S. enterica* isolates were *aadA2* and *SUL1*, each detected in 94.4% of isolates, followed by *bla*_CMY‐9_ (75%), *bla*_SHV_ (52.8%), *bla*_VIM_ (38.9%), and *bla*_TEM‐1_ (8.3%) (Figure 4 and Table S6). In contrast, no isolates carried quinolone resistance genes (*qnrA*, *qnrB*, *qnrC*, and *qnrS*) or several extended‐spectrum β‐lactamase (ESBL) genes (Figure 4 and Table S6).

The detection rate of resistance genes varied among poultry types, for example, *aadA2*/*SUL1* (layers: 92% vs. broilers: 100% vs. Sonali: 100%), *bla*_CMY‐9_ (76% vs. 66.7% vs. 100%), *bla*_SHV_ (56% vs. 33.3% vs. 100%), *bla*_VIM_ (40% vs. 22.2% vs. 100%), and *bla*_TEM‐1_ (12% vs. 0% vs. 0%) (Table S6). The same also occurred in different *S. enterica* serovars, for example, *aadA2*/*SUL1* (typhimurium: 100% vs. enteritidis: 80% vs. untyped: 100%), *bla*_CMY‐9_ (90.9% vs. 50% vs. 50%), *bla*_SHV_ (81.8% vs. 10% vs. 0%), *bla*_VIM_ (50% vs. 30% vs. 0%), and *bla*_TEM‐1_ (4.5% vs. 20% vs. 0%) (Table S6).

## 4 Discussion

This study provides crucial insights into the serovar‐specific distribution, virulence characteristics, and AMR profiles of *S. enterica* isolated from poultry in Bangladesh. The findings highlight the widespread occurrence of MDR and virulent *S. enterica* strains in commercial poultry systems, raising significant concerns for public health and food safety within the One Health framework. This study was conducted on small‐ to medium‐scale commercial poultry farms (broiler, layer, and Sonali) in four districts of Bangladesh. While our sampling provides valuable insights into *Salmonella* prevalence, virulence, and AMR in farm‐level poultry production, it does not encompass the entire commercial poultry sector of the country.

The overall prevalence of *S. enterica* in our study was 14.4%, aligning with prior reports from Bangladesh, which have shown varying rates depending on sampling method, poultry type, and geographic location. Studies by Borah et al. (2022), Siddiky et al. (2021), and Das et al. (2021) reported prevalence rates of 5.6%, 6.2%, and 24.6%, respectively. However, other studies from Bangladesh documented higher frequencies, particularly in flocks with clinical salmonellosis, for example, 70.1% by Sultana et al. (2021) and 78.2% by Sain et al. (2024). The variability in reported prevalence rates across different studies in Bangladesh may be attributed to several factors. Differences in sample size and study design can influence the robustness and generalizability of the findings, with smaller sample sets more prone to bias. Moreover, variation in analytical techniques, including culture‐based methods, PCR assays, and confirmatory tools such as MALDI‐TOF‐MS, may result in differences in sensitivity and specificity, thereby affecting prevalence estimates. Another possible factor is the prior use of antibiotics in poultry flocks, which could suppress bacterial growth and reduce the likelihood of detecting *Salmonella* during culture‐based assays. Together, these factors may explain the wide range of prevalence reported in Bangladesh and highlight the importance of methodological consistency in future surveillance studies. Moreover, other studies from different geographical locations detected a varied prevalence of *S. enterica* in chickens, for example, 21% by Osivand et al. (2025) and 8.94% by Piryaei et al. (2025) in Iran. The variation across studies may reflect differences in environmental contamination, biosecurity standards, and antimicrobial usage practices. The detection of *S. enterica* predominantly in birds exhibiting clinical signs, such as diarrhea, pasty vent, and hepatosplenomegaly, further underscores the pathogen's role in disease progression and flock morbidity.

Significant variation (*p* < 0.05) in prevalence was observed across poultry types, with layers showing the highest infection rate (25%), followed by broilers (9%) and Sonali chickens (4%). These findings align partially with previous studies in Bangladesh and South Africa, although prevalence trends vary by region. For instance, Siddiky et al. (2021) reported higher *Salmonella* prevalence in broilers and Sonali chickens in Bangladesh, while Sarker et al. (2021) in Bangladesh and Dlamini et al. (2021) in South Africa observed much higher rates in broilers compared with layers. Such discrepancies may be influenced by differences in biosecurity practices, farm management systems, and bird age and breed. Layers' prolonged lifespan and exposure time to potential environmental sources of contamination may partly explain their higher infection rates in our study. Sample type also influenced detection rates. Pooled liver tissue and gallbladder swabs yielded significantly more *S. enterica* isolates (23.8%) than cloacal swabs (10%). Similarly, Barac et al. (2024) and Fowler et al. (2021) reported higher *Salmonella* detection from internal tissues compared with fecal or cloacal samples. The high prevalence in pooled liver and gallbladder samples reinforces the systemic nature of infection in diseased birds and supports the selection of internal organs as more sensitive diagnostic sites for salmonellosis.

Globally, *S. enterica* serovars Typhimurium and Enteritidis are among the most frequently reported in poultry and humans (Dlamini et al. 2024). These serovars are also among the top causes of invasive NTS infections, particularly in developing countries (Hajra et al. 2023). Consistent with the global, and regional burden of specific serovars, *S. enterica* serovar Typhimurium (61.1%) and Enteritidis (27.8%) were predominant in our study, with untyped serovars comprising the remaining 11.1%. Typhimurium was most frequently detected in layers. This dominance is comparable to findings by Dlamini et al. (2024), Fowler et al. (2021), and Siddiky et al. (2021), who also highlighted Typhimurium and Enteritidis as the leading serovars in poultry. While some studies, such as Sain et al. (2024), reported a higher prevalence of *S. enterica* serovar Weltevreden, the predominance of Typhimurium and Enteritidis in our study suggests a continued zoonotic threat given their known association with human salmonellosis (Card et al. 2023). Moreover, the predominance of Typhimurium in our study aligns with reports from Southeast Asia and sub‐Saharan Africa, where it has been implicated in severe systemic infections and outbreaks (Kumar et al. 2025).

Generally, the pathogenicity of *Salmonella* relies on the interaction of numerous virulence factors, including the type III secretion systems encoded on SPIs, virulence plasmid factors, enterotoxin, and structural virulence factors, such as fimbriae and flagella (Shu et al. 2023). Salmonellosis results from various genes, including *invA*, *IpfA*, *hilA*, *sivH*, and *spvC*, which are considered major virulence genes (Siddiky et al. 2021). Moreover, virulence factors are essential for *S. enterica* colonization, systemic invasion, and immune evasion (Kaur and Jain 2012). In our study, all isolates harbored *invA*, *stn*, *sivH*, and *lpfA* genes, while *hilA* and *spvC* were detected in 97.2% and 63.9% of isolates, respectively. The universal detection of *invA* and *stn* aligns with previous studies (Sain et al. 2024; Siddiky et al. 2022; Elhariri et al. 2020), reinforcing their reliability as conserved molecular markers for *Salmonella* detection. The *invA* gene, encoding a component of the type III secretion system, plays a crucial role in epithelial cell invasion, while *stn* contributes to enterotoxin production and gastrointestinal symptoms (Naidoo et al. 2022; Oladapo et al. 2022). The *sivH* gene, involved in fimbrial production and Peyer's patch colonization, was also universally present and consistent with earlier findings in Bangladesh (Siddiky et al. 2022; Sain et al. 2024). The detection of *lpfA*, associated with long polar fimbriae and adhesion, in all isolates further confirms its essential role in host interaction. Similarly, *hilA*, a transcriptional regulator of invasion genes, was nearly universal in this study, as reported by Geyi et al. (2024) and Diab et al. (2023). Interestingly, the *spvC* gene, which contributes to intracellular survival and systemic dissemination, was present in 63.9% of isolates, lower than some international studies but consistent with Elhariri et al. (2020) and Das et al. (2022). The identification of virulence determinants in serovars Typhimurium and Enteritidis highlights their potential for severe disease and transmission through the food chain, posing a significant One Health concern (Ramatla et al. 2020).

The emergence and dissemination of AMR in pathogens such as NTS represent significant global challenges, particularly in low‐income countries (Beyene et al. 2024). Phenotypic antimicrobial susceptibility testing revealed widespread resistance to multiple antibiotic classes. All isolates were resistant to at least two agents, with 86.1% categorized as MDR. The high proportion of MDR *S. enterica* isolates observed in this study is a significant public health concern. This level is consistent with previous findings in Bangladesh poultry, where MDR prevalence ranged from 70% to 100% depending on serovar, sample type, and region (Siddiky et al. 2022; Das et al. 2022). Our data show that all untyped serovars, 81.8% of Typhimurium, and 90% of Enteritidis isolates exhibited MDR phenotypes, indicating widespread resistance regardless of serovar classification.

The highest resistance was observed for sulfamethoxazole (94.4%) and sulfonamide (91.7%), consistent with findings from Moraes et al. (2024), Vu et al. (2021), and Soares et al. (2023). Sulfonamide resistance often reflects the long‐term use of this drug class in veterinary practice. Resistance to nitrofurantoin (55.5%) in this study was moderate and similar to levels reported by Hassan et al. (2022), but higher than those observed in human clinical and environmental isolates (Yang et al. 2025; Rojas‐Sánchez et al. 2023). Resistance to streptomycin (52.8%) and ampicillin (52.8%) was also considerable. Although lower than values reported by Karabasanavar et al. (2022) and Chen et al. (2022), our findings suggest persistent resistance development due to their historical use in poultry. In contrast, resistance to nalidixic acid (13.9%), meropenem (8.3%), and ciprofloxacin (5.6%) was relatively low but still concerning, given the critical role of fluoroquinolones and carbapenems in treating invasive *Salmonella* infections (Kang et al. 2024; Zavari et al. 2025). Alarmingly, resistance to imipenem reached 50%, higher than several regional and global studies (Parvin et al. 2022; Sahu et al. 2025), suggesting emerging carbapenem resistance despite its restricted use in poultry. Cephalosporin resistance was also notable, with 33.3% of isolates resistant to ceftazidime and 13.9% to cefotaxime. These levels, though lower than in some pediatric and animal studies (Wu et al. 2021; Holohan et al. 2022), highlight the importance of monitoring ESBL–producing *Salmonella* in food animals. Resistance to amoxicillin‐clavulanate (8.3%) and ciprofloxacin was relatively low but consistent with prior Bangladeshi studies (Sarker et al. 2021; Talukder et al. 2023). Moreover, the calculated MAR index values ranged from 0.08 to 0.33, with several isolates exceeding the 0.2 threshold for high‐risk contamination sources (Elkenany et al. 2019). These values were lower than those reported by Siddiky et al. (2022) and Mir et al. (2022) but reflect significant exposure to diverse antimicrobial classes. The higher MAR index in untyped serovars and Sonali isolates may suggest environmental or management‐driven selection pressures.

Molecular‐based detection of resistance genes revealed high prevalence of *aadA2* (aminoglycoside resistance) and *SUL1* (sulfonamide resistance) genes, each detected in 94.4% of isolates. These findings are consistent with Wang et al. (2021) and highlight the potential for horizontal gene transfer via integrons or plasmids (Xu et al. 2020). Among β‐lactamase genes, *bla*_CMY‐9_ was the most prevalent (75%), followed by *bla*_SHV_ (52.8%) and *bla*_VIM_ (38.9%), whereas *bla*_TEM‐1_ was detected in only 8.3% of isolates. The predominance of *bla*_CMY‐9_ contrasts with some Bangladeshi and international studies where *bla*_TEM_ was more frequently observed (Das et al. 2022; Monira et al. 2017). The detection of *bla*_VIM_, a metallo‐β‐lactamase gene associated with carbapenem resistance, raises serious concerns about the emergence of carbapenemase‐producing *S. enterica* in poultry (Fernández et al. 2018; Huang et al. 2023). The absence of plasmid‐mediated quinolone resistance genes (*qnrA*, *qnrB*, *qnrC*, and *qnrS*) may suggest that fluoroquinolone resistance in these isolates is mediated by chromosomal mutations (e.g., gyrA/parC) or efflux pump mechanisms rather than plasmid‐associated genes, as documented in other studies (Robicsek et al. 2006; Rojas‐Sánchez et al. 2023). However, continued surveillance is necessary to detect future shifts in resistance mechanisms.

This study has some limitations, including a relatively small sample size from selected farms, a lack of stratified analysis of AMR by production type or geographical region, and the absence of correlation between phenotypic resistance and ARGs due to incomplete coverage of resistance genes. Nevertheless, it provides important baseline data on the prevalence, virulence, and AMR of *S. enterica* in Bangladeshi poultry farms, serving as a foundation for future large‐scale and longitudinal studies.

## 5 Conclusions

This study provides an updated and comprehensive assessment of *S. enterica* from poultry farms in Bangladesh, linking prevalence, serovars, virulence, and AMR patterns in a multidistrict, multiproduction setting. The predominance of serovars Typhimurium and Enteritidis, along with their high multidrug resistance rates and carriage of key virulence genes, reflects the potential risk these pathogens pose to animal and public health. The detection of resistance to critically important antimicrobials, including cephalosporins and carbapenems, is particularly concerning. These findings underscore the need for sustained surveillance, judicious antibiotic use in poultry production, and implementation of One Health‐based control strategies to mitigate the emergence and transmission of resistant *Salmonella* strains through the food chain.

## Author Contributions

**Najmun Nahar Popy:** conceptualization, investigation, writing – original draft, methodology, visualization, software, formal analysis, data curation. **Mohammad Ferdousur Rahman Khan:** conceptualization, funding acquisition, writing – review and editing, validation, project administration, resources, supervision. **Md. Saiful Islam:** writing – original draft, writing – review and editing, visualization, methodology, software, formal analysis, data curation. **Limon Biswas:** investigation, data curation. **Layla Yasmin:** investigation. **Mahbubul Pratik Siddique:** resources, writing – review and editing. **Marzia Rahman:** writing – review and editing, supervision. **Md. Bahanur Rahman:** conceptualization, writing – review and editing, funding acquisition, validation, project administration, supervision, resources.

## Ethics Statement

The authors have nothing to report.

## Conflicts of Interest

The authors declare that they have no known competing interests.

## Supporting information


**Supporting Table S1:** Primers used to detect *Salmonella enterica* and *Salmonella enterica* serovars. **Supporting Table S2:** Primers used for the detection of the virulence genes of *Salmonella enterica*. **Supporting Table S3:** Primers used for the detection of the resistance genes of *Salmonella enterica*. **Supporting Table S4:** Prevalence of virulence genes in *Salmonella* isolated from chickens in Bangladesh. **Supporting Table S5:** Antimicrobial resistance profiles of *Salmonella enterica* serovars isolated from poultry in Bangladesh. **Supporting Table S6:** Prevalence of antibiotic resistance genes in *Salmonella enterica* serovars isolated from poultry in Bangladesh.

## Data Availability

All the data are available in the manuscript and supporting information file.
