# Supplementary material for: Serovar‐Specific Antimicrobial Resistance and Virulence Profiles of Salmonella enterica From Poultry in Bangladesh
Source: Microbiologyopen. 2025 Oct 22;14(5):e70091. doi: 10.1002/mbo3.70091 (PMC12542807; doi:10.1002/mbo3.70091)
Supplement: Supplementary file 1 — Supporting Table S1: Primers used to detect Salmonella enterica and Salmonella enterica serovars. Supporting Table S2: Primers used for the detection of the virulence genes of Salmonella enterica. Supporting Table S3: Primers used for the detection of the resistance genes of Salmonella enterica. Supporting Table S4: Prevalence of virulence genes in Salmonella isolated from chickens in Bangladesh. Supporting Table S5: Antimicrobial resistance profiles of Salmonella enterica serovars isolated from poultry in Bangladesh. Supporting Table S6: Prevalence of antibiotic resistance genes in Salmonella enterica serovars isolated from poultry in Bangladesh. [file MBO3-14-e70091-s001.docx]

**Serovar-specific antimicrobial resistance and virulence profiles of *Salmonella enterica* from poultry in Bangladesh**

Najmun Nahar Popy^1^, Mohammad Ferdousur Rahman Khan^1^, Md. Saiful Islam^1,2^, Limon Biswas^1^, Layla Yasmin^1^, **Mahbubul Pratik Siddique^1^**, Marzia Rahman^1^, Md. Bahanur Rahman^1,*^

### ^1^ Department of Microbiology and Hygiene, Bangladesh Agricultural University, Mymensingh 2202, Bangladesh

^2^ Department of Animal Science, University of California – Davis, Davis 95616, United States

***Correspondence:**  [bahanurr@bau.edu.bd](mailto:bahanurr@bau.edu.bd)

**Supplementary Table S1:** Primers used to detect *Salmonella enterica* and *Salmonella enterica* serovars.

| Targeted Serovar | Prime Name | Sequence | Amplicon  Size (bp) | Reference |
| --- | --- | --- | --- | --- |
| *Salmonella enterica* | *bcfC*-F  *bcfC*-R | GGG TGG GCG GAA AAC TAT TTC  CGG CAC GGC GGA ATA GAG CAC | 993 | Salazar et al., 2019 |
| *Salmonella*  Typhimurium | *fliC-F*  *fliC-R* | CGG TGT TGC CCA GGT TGG TAA T  ACT GGT AAA GAT GGCT | 620 | Salazar et al., 2019 |
| *Salmonella*  Enteritidis | *sdf*-F  *sdf*-R | TGTGTTTTATCTGATGCAAGAGG  CGTTCTTCTGGTACTTACGATGAC | 333 | Rezaei et al., 2022 |

**Supplementary Table S2:** Primers used for the detection of the virulence genes of *Salmonella enterica*

| **Virulence Factor** | **Target gene** | **Sequence** | **Amplicon Size (bp)** | **Reference** |
| --- | --- | --- | --- | --- |
| Invasion | *invA* | F-CGGTGGTTTTAAGCGTACTCTT  R- CGAATATGCTCCACAAGGTTA | 796 | Arkali and Çetinkaya, 2020 |
| Plasmid | *spvC* | F- ACTCCTTGCACAACCAAATGCGGA  R-TGTCTTCTGCATTTCGCCACCAATCA | 571 | Yulian et al., 2020 |
| Enterotoxin | *stn* | F-TATTTTGCACCACAGCCAGC  R-CGACCGCGTTATCATCACTG | 131 | Farhat et al., 2023 |
| Fimbriae | *lpfA* | F-CTTTCGCTGCTGAATCTGGT  R-CAGTGTTAACAGAAACCAGT | 250 | Siddiky et al., 2022 |
| Regulatory protein | *hilA* | F-CTGCCGCAGTGTTAAGGATA  R-CTGTCGCCTTAATCGCATGT | 497 | Lozano-Villegas et al., 2023 |
| Effector protein | *sivH* | F-GTATGCGAACAAGCGTAACAC  R-CAGAATGCGAATCCTTCGCAC | 763 | Ndlovu et al., 2023 |

**Supplementary Table S3:** Primers used for the detection of the resistance genes of *Salmonella enterica*

| **Target gene** | **Sequence (5´-3´)** | **Annealing Temperature (ᵒC)** | **PCR product Size(bp)** | **Reference** |
| --- | --- | --- | --- | --- |
| *bla*_TEM_ | F-CAG CGG TAA GAT CCT TGA GA  R- ACT CCC CGT CGT GTA GAT AA | 55 | 643 | Chen et al., 2004 |
| *bla*_NDM_ | F- CACCTCATGTTTGAATTCGCC  R- CTCTGTCACATCGAAATCGC | 58 | 984 | Odewale et al., 2023 |
| *bla*_IMP_ | F- CTACCGCAGCGAGTCTTTG  R- AACCAGTTTTGCCTTACCAT | 55 | 587 | Li et al., 2022 |
| *BLA_VIM_* | F ‑GTGCTTTGACAACGTTCGCT  R ‑TCCACGCACTTTCATGACGA | 58 | 442 | Kanaan et al., 2022 |
| *BLA_SHV_* | F-GGG TTA TTC TTA TTT GTC GC  R-TTA GCG TTG CCA GTG CTC | 58 | 567 | Warjri et al., 2015 |
| *BLA_CTXM_* | F- ACGCTGTTGTTAGGAAGTG  R- TTG AGG CTG GGT GAA GT | 58 | 759 | Warjri et al., 2015 |
| *BLA_CMY2_* | F-TGG CCG TTG CCG TTA TCT AC  R-CCC GTT TTA TGC ACC CAT GA | 55 | 870 | Chen et al., 2004 |
| *BLA_CMY9_* | F-TCA GCG AGC AGA CCC TGT TC  R-CTG GCC GGG ATG GGA TAG TT | 55 | 874 | Chen et al., 2004 |
| *BLA_CTXM1_* | F- AAC CGT CAC GCT GTT GTT AG  R- TTG AGG CTG GGT GAA GTA AG | 55 | 766 | Chen et al., 2004 |
| *BLA_CTXM2_* | F-GGC GTT GCG CTG ATT AAC AC  R-TTG CCC TTA AGC CAC GTC AC | 55 | 486 | Chen et al., 2004 |
| *bla_CTXM14_* | F- GCC TGC CGA TCT GGT TAA CT  R- GCC GGT CGT ATT GCC TTT GA | 55 | 358 | Chen et al., 2004 |
| *bla_OXA_* | F- ATATCTCTACTGTTGCATCTCC  R- AAACCCTTCAAACCATCC | 48 | 619 | Chen et al., 2004 |
| *bla_OXA_* | F- ACACAATACATATCAACTTCGC  R- AGTGTGTTTAGAATGGTGATC | 58 | 813 | Kuang et al., 2018 |
| *qnrA* | F-GGGTATGGATATTATTGATAAAG  R-CTAATCCGGCAGCACTATTTA | 54 | 670 | Veldman et al., 2008 |
| *qnrB* | F-ATGACGCCATTACTGTATAA  R- GATCGCAATGTGTGAAGTTT | 53 | 678 | Veldman et al., 2008 |
| *qnrC* | F-GGGTTGTACATTTATTGAATC  R- TCCACTTTACGAGGTTCT | 52 | 447 | Xu et al., 2015 |
| *qnrS* | F- GCAAGTTCATTGAACAGGGT  R- TCTAAACCGTCGAGTTCGGCG | 54 | 428 | El-Shazly et al., 2015 |
| *aadA2* | F-TGTTGGTTACTGTGGCCGTA  R-GATCTCGCCTTTCACAAAGC | 60 | 623 | Walker et al., 2001 |
| *sul1* | F-TCACCGAGGACTCCTTCTTC  R-AATATCGGGATAGAGCGCAG | 60 | 316 | Randall et al., 2004 |

**Supplementary Table S4.** Prevalence of virulence genes in *Salmonella* isolated from chickens in Bangladesh

| **Virulence genes** | **Poultry types** | | | ***Salmonella enterica* serovars** | | | **Overall (n = 36)** |
| --- | --- | --- | --- | --- | --- | --- | --- |
|  | **Layers (n = 25)** | **Broilers (n = 9)** | **Sonali (n = 2)** | **Typhimurium (n = 22)** | **Enteritidis (n = 10)** | **Untyped (n = 4)** |  |
| *invA* | 25^a^ (100%, 86.7-100) | 9^a^ (100%, 70.1-100) | 2^a^ (100%, 17.8-100) | 22^a^ (100%, 85.1-100) | 10^a^ (100%, 72.3-100) | 4^a^ (100%, 51.0-100) | 36 (100%, 90.4-100) |
| *stn* | 25^a^ (100%, 86.7-100) | 9^a^ (100%, 70.1-100) | 2^a^ (100%, 17.8-100) | 22^a^ (100%, 85.1-100) | 10^a^ (100%, 72.3-100) | 4^a^ (100%, 51.0-100) | 36 (100%, 90.4-100) |
| *spvC* | 18^a^ (72%, 52.4-85.7) | 5^a^ (55.6%, 26.7-81.1) | 0^a^ (0%, 0-82.2) | 15^a^ (68.2%, 47.3-83.6) | 5^a^ (50%, 23.7-76.3) | 3^a^ (75%, 30.1-98.7) | 23 (63.9%, 47.6-77.5) |
| *hilA* | 25^a^ (100%, 86.7-100) | 8^a^ (88.9%, 56.5-99.4) | 2^a^ (100%, 17.8-100) | 22^a^ (100%, 85.1-100) | 9^a^ (90%, 59.6-99.5) | 4^a^ (100%, 51.0-100) | 35 (97.2%, 85.8-99.9) |
| *sivH* | 25^a^ (100%, 86.7-100) | 9^a^ (100%, 70.1-100) | 2^a^ (100%, 17.8-100) | 22^a^ (100%, 85.1-100) | 10^a^ (100%, 72.3-100) | 4^a^ (100%, 51.0-100) | 36 (100%, 90.4-100) |
| *lpfA* | 25^a^ (100%, 86.7-100) | 9^a^ (100%, 70.1-100) | 2^a^ (100%, 17.8-100) | 22^a^ (100%, 85.1-100) | 10^a^ (100%, 72.3-100) | 4^a^ (100%, 51.0-100) | 36 (100%, 90.4-100) |

**Supplementary Table S5.** Antimicrobial resistance profiles of *Salmonella enterica* serovars isolated from poultry in Bangladesh

| **Antibiotic classes** | **Name of antibiotics** | **Poultry types** | | | ***Salmonella enterica* serovars** | | | **Overall (n = 36)** |
| --- | --- | --- | --- | --- | --- | --- | --- | --- |
|  |  | **Layers (n = 25)** | **Broilers (n = 9)** | **Sonali (n = 2)** | **Typhimurium (n = 22)** | **Enteritidis (n = 10)** | **Untyped (n = 4)** |  |
| Penicillins | AMP | 13^a^ (52%, 33.5-69.9) | 5^a^ (55.6%, 26.7-81.1) | 1^a^ (50%, 2.6-97.4) | 8^a^ (36.4%, 19.7-57.0) | 7^a^ (70%, 39.7-89.2) | 4^a^ (100%, 51.0-100) | 19 (52.8%, 37.0-68.0) |
| Carbapenems | MEM | 1^a^ (4%, 0.2-19.5) | 2^a^ (22.2%, 3.9-54.7) | 0^a^ (0%, 0-82.2) | 0^a^ (0%, 0-14.9) | 2^a^ (20%, 3.6-50.9) | 1^a^ (25%, 1.3-69.9) | 3 (8.3%, 2.9-21.8) |
|  | IMP | 15^a^ (60%, 40.7-76.6) | 3^a^ (33.3%, 12.1-64.6) | 0^a^ (0%, 0-82.2) | 13^a^ (59.1%, 38.7-76.7) | 5^a^ (50%, 23.7-76.3) | 0^a^ (0%, 0-48.9) | 18 (50%, 34.5-65.5) |
| Cephalosporins | CTR | 0^a^ (0%, 0-13.3) | 0^a^ (0%, 0-29.9) | 0^a^ (0%, 0-82.2) | 0^a^ (0%, 0-14.9) | 0^a^ (0%, 0-27.8) | 0^a^ (0%, 0-48.9) | 0 (0%, 0-9.6) |
|  | CAZ | 9^a,b^ (36%, 20.2-55.5) | 1^b^ (11.1%, 0.6-43.5) | 2^a^ (100%, 17.8-100) | 9^a^ (40.9%, 23.3-61.3) | 2^a^ (20%, 3.6-50.9) | 1^a^ (25%, 1.3-69.9) | 12 (33.3%, 20.2-49.7) |
|  | CTX | 4^a^ (16%, 6.4-34.7) | 1^a^ (11.1%, 0.6-43.5) | 0^a^ (0%, 0-82.2) | 4^a^ (18.2%, 7.3-38.5) | 0^a^ (0%, 0-27.8) | 1^a^ (25%, 1.3-69.9) | 5 (13.9%, 6.1-28.7) |
|  | CPM | 0^a^ (0%, 0-13.3) | 0^a^ (0%, 0-29.9) | 0^a^ (0%, 0-82.2) | 0^a^ (0%, 0-14.9) | 0^a^ (0%, 0-27.8) | 0^a^ (0%, 0-48.9) | 0 (0%, 0-9.6) |
| Beta-lactam inhibitors | AMC | 2^a^ (8%, 1.4-24.9) | 1^a^ (11.1%, 0.6-43.5) | 0^a^ (0%, 0-82.2) | 1^a^ (4.5%, 0.2-21.8) | 1^a^ (10%, 0.5-40.4) | 1^a^ (25%, 1.3-69.9) | 3 (8.3%, 2.9-21.8) |
| Aminoglycosides | GEN | 0^a^ (0%, 0-13.3) | 0^a^ (0%, 0-29.9) | 0^a^ (0%, 0-82.2) | 0^a^ (0%, 0-14.9) | 0^a^ (0%, 0-27.8) | 0^a^ (0%, 0-48.9) | 0 (0%, 0-9.6) |
|  | AK | 0^a^ (0%, 0-13.3) | 0^a^ (0%, 0-29.9) | 0^a^ (0%, 0-82.2) | 0^a^ (0%, 0-14.9) | 0^a^ (0%, 0-27.8) | 0^a^ (0%, 0-48.9) | 0 (0%, 0-9.6) |
|  | S | 9^a^ (36%, 20.2-55.5) | 8^b^ (88.9%, 56.5-99.4) | 2^a,b^ (100%, 17.8-100) | 11^a^ (50%, 30.7-69.3) | 5^a^ (50%, 23.7-76.3) | 3^a^ (75%, 30.1-98.7) | 19 (52.8%, 37.0-68.0) |
| Quinolones | CIP | 2^a^ (8%, 1.4-24.9) | 0^a^ (0%, 0-29.9) | 0^a^ (0%, 0-82.2) | 1^a^ (4.5%, 0.2-21.8) | 1^a^ (10%, 0.5-40.4) | 0^a^ (0%, 0-48.9) | 2 (5.6%, 0.9-18.1) |
|  | LE | 0^a^ (0%, 0-13.3) | 0^a^ (0%, 0-29.9) | 0^a^ (0%, 0-82.2) | 0^a^ (0%, 0-14.9) | 0^a^ (0%, 0-27.8) | 0^a^ (0%, 0-48.9) | 0 (0%, 0-9.6) |
|  | NA | 3^a^ (12%, 4.2-29.9) | 2^a^ (22.2%, 3.9-54.7) | 0^a^ (0%, 0-82.2) | 1^a^ (4.5%, 0.2-21.8) | 4^b^ (40%, 16.8-68.7) | 0^a,b^ (0%, 0-48.9) | 5 (13.9%, 6.1-28.7) |
| Tetracyclines | TE | 0^a^ (0%, 0-13.3) | 0^a^ (0%, 0-29.9) | 0^a^ (0%, 0-82.2) | 0^a^ (0%, 0-14.9) | 0^a^ (0%, 0-27.8) | 0^a^ (0%, 0-48.9) | 0 (0%, 0-9.6) |
|  | DO | 0^a^ (0%, 0-13.3) | 0^a^ (0%, 0-29.9) | 0^a^ (0%, 0-82.2) | 0^a^ (0%, 0-14.9) | 0^a^ (0%, 0-27.8) | 0^a^ (0%, 0-48.9) | 0 (0%, 0-9.6) |
| Folate pathway antagonists | COT | 0^a^ (0%, 0-13.3) | 0^a^ (0%, 0-29.9) | 0^a^ (0%, 0-82.2) | 0^a^ (0%, 0-14.9) | 0^a^ (0%, 0-27.8) | 0^a^ (0%, 0-48.9) | 0 (0%, 0-9.6) |
|  | S3 | 23^a^ (92%, 75.0-98.6) | 8^a^ (88.9%, 56.5-99.4) | 2^a^ (100%, 17.8-100) | 22^a^ (100%, 85.1-100) | 7^b^ (70%, 39.7-89.2) | 4^a,b^ (100%, 51.0-100) | 33 (91.7%, 78.2-97.1) |
|  | SMX | 23^a^ (92%, 75.0-98.6) | 9^a^ (100%, 70.1-100) | 2^a^ (100%, 17.8-100) | 22^a^ (100%, 85.1-100) | 8^a^ (80%, 49.0-96.4) | 4^a^ (100%, 51.0-100) | 34 (94.4%, 81.9-99.0) |
|  | TM | 0^a^ (0%, 0-13.3) | 0^a^ (0%, 0-29.9) | 0^a^ (0%, 0-82.2) | 0^a^ (0%, 0-14.9) | 0^a^ (0%, 0-27.8) | 0^a^ (0%, 0-48.9) | 0 (0%, 0-9.6) |
| Monobactams | AT | 0^a^ (0%, 0-13.3) | 0^a^ (0%, 0-29.9) | 0^a^ (0%, 0-82.2) | 0^a^ (0%, 0-14.9) | 0^a^ (0%, 0-27.8) | 0^a^ (0%, 0-48.9) | 0 (0%, 0-9.6) |
| Macrolides | AZM | 0^a^ (0%, 0-13.3) | 0^a^ (0%, 0-29.9) | 0^a^ (0%, 0-82.2) | 0^a^ (0%, 0-14.9) | 0^a^ (0%, 0-27.8) | 0^a^ (0%, 0-48.9) | 0 (0%, 0-9.6) |
| Phenicols | FOS | 0^a^ (0%, 0-13.3) | 0^a^ (0%, 0-29.9) | 0^a^ (0%, 0-82.2) | 0^a^ (0%, 0-14.9) | 0^a^ (0%, 0-27.8) | 0^a^ (0%, 0-48.9) | 0 (0%, 0-9.6) |
| Nitrofurans | F | 12^a^ (48%, 30.0-66.5) | 6^a^ (66.7%, 35.4-87.9) | 2^a^ (100%, 17.8-100) | 12^a^ (54.5%, 34.7-73.1) | 6^a^ (60%, 31.3-83.2) | 2^a^ (50%, 8.9-91.1) | 20 (55.6%, 39.6-70.5) |

Here, AMP = Ampicillin, MEM = Meropenem, IMP = Imipenem, CTR = Ceftriaxone, CAZ = Ceftazidime, CTX = Cefotaxime, CPM = Cefepime, AMC = Amoxicillin-clavulanate, GEN = Gentamicin, AK = Amikacin, S = Streptomycin, CIP = Ciprofloxacin, LE = Levofloxacin, NA = Nalidixic Acid, TE = Tetracycline, DO = Doxycycline, COT = Trimethoprim-sulfamethoxazole, S3 = Sulfonamide, SMX = Sulfamethoxazole, TM = Sulfamethoxazole, AT = Aztreonam, AZM = Azithromycin, FOS = Fosfomycin, F = Nitrofurantoin.

**Supplementary Table S6.** Prevalence of antibiotic resistance genes in *Salmonella enterica* serovars isolated from poultry in Bangladesh

| **Name of groups** | **Name of resistance genes** | **Poultry types** | | | ***Salmonella enterica* serovars** | | | **Overall (n = 36)** |
| --- | --- | --- | --- | --- | --- | --- | --- | --- |
|  |  | **Layers (n = 25)** | **Broilers (n = 9)** | **Sonali (n = 2)** | **Typhimurium (n = 22)** | **Enteritidis (n = 10)** | **Untyped (n = 4)** |  |
| Beta-lactams | *bla*_TEM_ | 0^a^ (0%, 0-13.3) | 0^a^ (0%, 0-29.9) | 0^a^ (0%, 0-82.2) | 0^a^ (0%, 0-14.9) | 0^a^ (0%, 0-27.8) | 0^a^ (0%, 0-48.9) | 0 (0%, 0-9.6) |
|  | *bla*_TEM-1_ | 3^a^ (12%, 4.2-29.9) | 0^a^ (0%, 0-29.9) | 0^a^ (0%, 0-82.2) | 1^a^ (4.5%, 0.2-21.8) | 2^a^ (20%, 3.6-50.9) | 0^a^ (0%, 0-48.9) | 3 (8.3%, 2.9-21.8) |
|  | *bla*_NDM_ | 0^a^ (0%, 0-13.3) | 0^a^ (0%, 0-29.9) | 0^a^ (0%, 0-82.2) | 0^a^ (0%, 0-14.9) | 0^a^ (0%, 0-27.8) | 0^a^ (0%, 0-48.9) | 0 (0%, 0-9.6) |
|  | *bla*_IMP_ | 0^a^ (0%, 0-13.3) | 0^a^ (0%, 0-29.9) | 0^a^ (0%, 0-82.2) | 0^a^ (0%, 0-14.9) | 0^a^ (0%, 0-27.8) | 0^a^ (0%, 0-48.9) | 0 (0%, 0-9.6) |
|  | *bla*_VIM_ | 10^a^ (40%, 23.4-59.3) | 2^a^ (22.2%, 3.9-54.7) | 2^a^ (100%, 17.8-100) | 11^a^ (50%, 30.7-69.3) | 3^a^ (30%, 10.8-60.3) | 0^a^ (0%, 0-48.9) | 14 (38.9%, 24.8-55.1) |
|  | *bla*_SHV_ | 14^a^ (56%, 37.1-73.3) | 3^a^ (33.3%, 12.1-64.6) | 2^a^ (100%, 17.8-100) | 18^a^ (81.8%, 61.5-92.7) | 1^b^ (10%, 0.5-40.4) | 0^b^ (0%, 0-48.9) | 19 (52.8%, 37.0-68.0) |
|  | *bla*_CMY-2_ | 0^a^ (0%, 0-13.3) | 0^a^ (0%, 0-29.9) | 0^a^ (0%, 0-82.2) | 0^a^ (0%, 0-14.9) | 0^a^ (0%, 0-27.8) | 0^a^ (0%, 0-48.9) | 0 (0%, 0-9.6) |
|  | *bla*_CMY-9_ | 19^a^ (76%, 56.6-88.5) | 6^a^ (66.7%, 35.4-87.9) | 2^a^ (100%, 17.8-100) | 20^a^ (90.9%, 72.2-98.4) | 5^b^ (50%, 23.7-76.3) | 2^a,b^ (50%, 8.9-91.1) | 27 (75%, 58.9-86.3) |
|  | *bla*_CTX-M_ | 0^a^ (0%, 0-13.3) | 0^a^ (0%, 0-29.9) | 0^a^ (0%, 0-82.2) | 0^a^ (0%, 0-14.9) | 0^a^ (0%, 0-27.8) | 0^a^ (0%, 0-48.9) | 0 (0%, 0-9.6) |
|  | *bla*_CTX-M-1_ | 0^a^ (0%, 0-13.3) | 0^a^ (0%, 0-29.9) | 0^a^ (0%, 0-82.2) | 0^a^ (0%, 0-14.9) | 0^a^ (0%, 0-27.8) | 0^a^ (0%, 0-48.9) | 0 (0%, 0-9.6) |
|  | *bla*_CTX-M-2_ | 0^a^ (0%, 0-13.3) | 0^a^ (0%, 0-29.9) | 0^a^ (0%, 0-82.2) | 0^a^ (0%, 0-14.9) | 0^a^ (0%, 0-27.8) | 0^a^ (0%, 0-48.9) | 0 (0%, 0-9.6) |
|  | *bla*_CTX-M-14_ | 0^a^ (0%, 0-13.3) | 0^a^ (0%, 0-29.9) | 0^a^ (0%, 0-82.2) | 0^a^ (0%, 0-14.9) | 0^a^ (0%, 0-27.8) | 0^a^ (0%, 0-48.9) | 0 (0%, 0-9.6) |
|  | *bla*_OXA-1_ | 0^a^ (0%, 0-13.3) | 0^a^ (0%, 0-29.9) | 0^a^ (0%, 0-82.2) | 0^a^ (0%, 0-14.9) | 0^a^ (0%, 0-27.8) | 0^a^ (0%, 0-48.9) | 0 (0%, 0-9.6) |
| Quinolones | *qnrA* | 0^a^ (0%, 0-13.3) | 0^a^ (0%, 0-29.9) | 0^a^ (0%, 0-82.2) | 0^a^ (0%, 0-14.9) | 0^a^ (0%, 0-27.8) | 0^a^ (0%, 0-48.9) | 0 (0%, 0-9.6) |
|  | *qnrB* | 0^a^ (0%, 0-13.3) | 0^a^ (0%, 0-29.9) | 0^a^ (0%, 0-82.2) | 0^a^ (0%, 0-14.9) | 0^a^ (0%, 0-27.8) | 0^a^ (0%, 0-48.9) | 0 (0%, 0-9.6) |
|  | *qnrC* | 0^a^ (0%, 0-13.3) | 0^a^ (0%, 0-29.9) | 0^a^ (0%, 0-82.2) | 0^a^ (0%, 0-14.9) | 0^a^ (0%, 0-27.8) | 0^a^ (0%, 0-48.9) | 0 (0%, 0-9.6) |
|  | *qnrS* | 0^a^ (0%, 0-13.3) | 0^a^ (0%, 0-29.9) | 0^a^ (0%, 0-82.2) | 0^a^ (0%, 0-14.9) | 0^a^ (0%, 0-27.8) | 0^a^ (0%, 0-48.9) | 0 (0%, 0-9.6) |
| Aminoglycosides | *aadA2* | 23^a^ (92%, 75.0-98.6) | 9^a^ (100%, 70.1-100) | 2^a^ (100%, 17.8-100) | 22^a^ (100%, 85.1-100) | 8^a^ (80%, 49.0-96.4) | 4^a^ (100%, 51.0-100) | 34 (94.4%, 81.9-99.0) |
| Folate pathway antagonists | *SUL-1* | 23^a^ (92%, 75.0-98.6) | 9^a^ (100%, 70.1-100) | 2^a^ (100%, 17.8-100) | 22^a^ (100%, 85.1-100) | 8^a^ (80%, 49.0-96.4) | 4^a^ (100%, 51.0-100) | 34 (94.4%, 81.9-99.0) |

**References**

Arkali, A. and Çetinkaya, B., 2020. Molecular identification and antibiotic resistance profiling of Salmonella species isolated from chickens in eastern Turkey. BMC veterinary research, 16(1), p.205.

Chen S, Zhao S, White DG, Schroeder CM, Lu R, Yang H, et al. Characterization of multiple-antimicrobial-resistant Salmonella serovars isolated from retail meats. Applied and Environmental Microbiology. 2004;70(1):1-7.

El-Shazly S, Dashti A, Vali L, Bolaris M, Ibrahim AS. Molecular epidemiology and characterization of multiple drug-resistant (MDR) clinical isolates of Acinetobacter baumannii. International Journal of Infectious Diseases. 2015;41:42-9.

Farhat, M., Khayi, S., Berrada, J., Mouahid, M., Ameur, N., El-Adawy, H. and Fellahi, S., 2023: Salmonella enterica Serovar Gallinarum Biovars Pullorum and Gallinarum in Poultry: Review of Pathogenesis, Antibiotic Resistance, Diagnosis and Control in the Genomic Era. Antibiotics 13 23.

Kanaan MHG, Khalil ZK, Khashan HT, Ghasemian A. Occurrence of virulence factors and carbapenemase genes in Salmonella enterica serovar Enteritidis isolated from chicken meat and egg samples in Iraq. BMC microbiology. 2022;22(1):279.

Kuang D, Zhang J, Xu X, Shi W, Yang X, Su X, et al. Increase in ceftriaxone resistance and widespread extended-spectrum β-lactamases genes among Salmonella enterica from human and nonhuman sources. Foodborne Pathogens and Disease. 2018;15(12):770-5.

Li Y, Kang X, Ed-Dra A, Zhou X, Jia C, Müller A, et al. Genome-based assessment of antimicrobial resistance and virulence potential of isolates of non-pullorum/gallinarum Salmonella Serovars recovered from dead poultry in China. Microbiology spectrum. 2022;10(4):e00965-22.

Lozano-Villegas, K.J., Herrera-Sánchez, M.P., Beltrán-Martínez, M.A., Cárdenas-Moscoso, S. and Rondón-Barragán, I.S., 2023. Molecular detection of virulence factors in Salmonella serovars isolated from poultry and human samples. Veterinary Medicine International, 2023(1), p.1875253.

Ndlovu, L., Butaye, P., Maliehe, T.S., Magwedere, K., Mankonkwana, B.B., Basson, A.K., Ngema, S.S. and Madoroba, E., 2023: Virulence and antimicrobial resistance profiling of Salmonella serovars recovered from retail poultry offal in KwaZulu-Natal province, South Africa. Pathogens 12 641.

Odewale G, Jibola-Shittu MY, Ojurongbe O, Olowe RA, Olowe OA. Genotypic determination of Extended Spectrum β-Lactamases and carbapenemase production in clinical isolates of Klebsiella pneumoniae in Southwest Nigeria. Infectious Disease Reports. 2023;15(3):339-53.

Randall L, Cooles S, Osborn M, Piddock L, Woodward MJ. Antibiotic resistance genes, integrons and multiple antibiotic resistance in thirty-five serotypes of Salmonella enterica isolated from humans and animals in the UK. Journal of Antimicrobial Chemotherapy. 2004;53(2):208-16.

Rezaei A, Hashemi FB, Heshteli RR, Rahmani M, Halimi S. Frequency of Salmonella serotypes among children in Iran: Antimicrobial susceptibility, biofilm formation, and virulence genes. BMC pediatrics. 2022;22(1):557.

Salazar GA, Guerrero-López R, Lalaleo L, Avilés-Esquivel D, Vinueza-Burgos C, Calero-Cáceres W. Presence and diversity of Salmonella isolated from layer farms in central Ecuador. F1000Research. 2019;8.

Siddiky NA, Sarker S, Khan SR, Rahman T, Kafi A, Samad MA (2022) Virulence and antimicrobial resistance profile of non-typhoidal Salmonella enterica serovars recovered from poultry processing environments at wet markets in Dhaka, Bangladesh. PLoS ONE 17(2): e0254465.

Veldman K, van Pelt W, Mevius D. First report of qnr genes in Salmonella in The Netherlands. Journal of Antimicrobial Chemotherapy. 2008;61(2):452-3.

Walker RA, Lindsay E, Woodward MJ, Ward LR, Threlfall EJ. Variation in clonality and antibiotic-resistance genes among multiresistant Salmonella enterica serotype typhimurium phage-type U302 (MR U302) from humans, animals, and foods. Microbial Drug Resistance. 2001;7(1):13-21.

Warjri I, Dutta T, Lalzampuia H, Chandra R. Detection and characterization of extended-spectrum β-lactamases (blaCTX-M-1 and blaSHV) producing Escherichia coli, Salmonella spp. and Klebsiella pneumoniae isolated from humans in Mizoram. Veterinary world. 2015;8(5):599.

Xu G, An W, Wang H, Zhang X. Prevalence and characteristics of extended-spectrum β-lactamase genes in Escherichia coli isolated from piglets with post-weaning diarrhea in Heilongjiang province, China. Frontiers in microbiology. 2015;6:1103.

Yulian, R., Narulita, E., Iqbal, M., Sari, D.R., Suryaningsih, I. and Ningrum, D.E.A.F., 2020: Detection of virulence and specific genes of Salmonella sp. indigenous from Jember, Indonesia. Biodiversitas 21 2889-2892.
